# Molecular Regulation of the Mitochondrial F_**1**_F_**o**_-ATPsynthase: Physiological and Pathological Significance of the Inhibitory Factor 1 (IF_**1**_)

**DOI:** 10.1155/2012/367934

**Published:** 2012-08-26

**Authors:** Danilo Faccenda, Michelangelo Campanella

**Affiliations:** ^1^The Royal Veterinary College, University of London and UCL Consortium for Mitochondrial Research, Royal College Street, London NW1 0TU, UK; ^2^European Brain Research Institute, Rita Levi-Montalcini Foundation, 00143 Rome, Italy

## Abstract

In mammals, the mitochondrial F_1_F_o_-ATPsynthase sets out the energy homeostasis by producing the bulk of cellular ATP. As for every enzyme, the laws of thermodynamics command it; however, it is privileged to have a dedicated molecular regulator that controls its rotation. This is the so-called ATPase Inhibitory Factor 1 (IF_1_) that blocks its reversal to avoid the consumption of cellular ATP when the enzyme acts as an ATP hydrolase. Recent evidence has also demonstrated that IF_1_ may control the alignment of the enzyme along the mitochondrial inner membrane, thus increasing the interest for the molecule. We conceived this review to outline the fundamental knowledge of the F_1_F_o_-ATPsynthase and link it to the molecular mechanisms by which IF_1_ regulates its way of function, with the ultimate goal to highlight this as an important and possibly unique means to control this indispensable enzyme in both physiological and pathological settings.

## 1. Introduction

The F_1_F_o_-ATPsynthase is an H^+^-pumping ATPase evolutionary specialized in synthesizing ATP by using an H^+^ gradient generated across a biological membrane. It is present in bacteria and intracellular organelles such as chloroplasts and mitochondria. In these, the enzyme is hosted within the inner membrane as part of the OXPHOS, where it couples the transport of H^+^ from the intermembrane space into the matrix with the synthesis of ATP, guaranteeing the supply of energy to biological processes, since the majority of cellular ATP is generated by the mitochondrial F_1_F_o_-ATPsynthase.

The molecular structure, catalytic mechanism, and regulation of the mitochondrial F_1_F_o_-ATPsynthase were described by the seminal work of the Nobel Laureates Mitchell, Boyer and Walker, that revealed its complexity and the functional steps that drive the synthesis of ATP.

Besides its role as energy producer, the mitochondrial F_1_F_o_-ATPsynthase is also essential for the maintenance of the mitochondrial membrane potential (ΔΨ_*m*_) [1]—which is crucial for the import of proteins into the organelle [2]—and cristae structure [3]. In addition, it is fundamental for an optimal supramolecular organization of the respiratory chain [4], and for regulating the mobilization of cytochrome *c* during apoptosis [5].

In animals and plants, the mitochondrial F_1_F_o_-ATPsynthase is molecularly regulated by an endogenous, nuclear-encoded polypeptide, the ATPase Inhibitory Factor 1 (IF_1_). IF_1_ is primarily responsible for inhibiting the hydrolysis of ATP by the ATP synthase [6], an event that occurs when the electrochemical proton gradient across the mitochondrial inner membrane is lost (e.g., during hypoxic/ischaemic conditions), and the enzyme reverses to restore ΔΨ_*m*_ [7]. A number of studies have shown that IF_1_ is also involved in the regulation of the oligomeric state of the F_1_F_o_-ATPsynthase, by facilitating the enzyme's dimerization via a molecular link between two F_1_ domains [8]; for this reason, it is also implicated in the remodelling of cristae structure [9], and consequently in the regulation of mito-ultrastructure and morphology.

The interest for this inhibitor, or regulator—as we like to consider it—stems from many reasons; among them, the evidence for a key role in pathologies is the most meaningful but the less explored. IF_1_ overexpression is reported in human carcinomas [10], differences in the ratio of expression between IF_1_ and the F_1_F_o_-ATPsynthase are related to changes in cellular responses to ischaemia/reperfusion injury [11, 12], and its absence is recorded in a rare form of mitochondrial myopathy called Luft's disease [13, 14].

Despite this, IF_1_ seems underconsidered in pathologies whose etiology correlates with defective mitochondrial F_1_F_o_-ATPsynthase. Here, we will explain why the interaction between IF_1_ and F_1_F_o_-ATPsynthase is important, and why the quality of cellular bioenergetics depends on it.

## 2. Molecular Structure and Catalysis of the **** F_1_F_o_-ATPsynthase 

**Figure 1:**
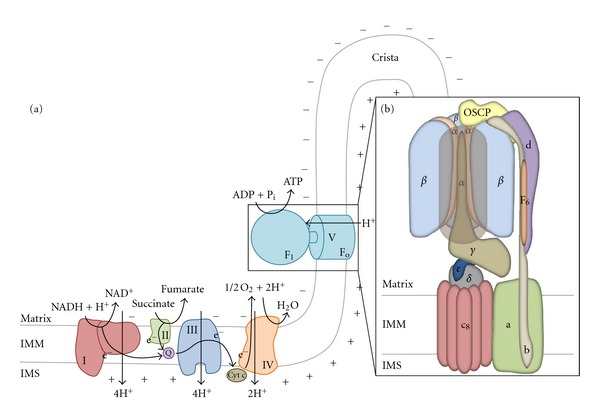
Oxidative phosphorylation and the mammalian F_1_F_o_-ATPsynthase. (a) Scheme of the mitochondrial OXPHOS: it is composed of five complexes, which couple the generation of a proton motive force through the mitochondrial inner membrane (IMM) with ATP synthesis. The first four complexes form the electron-transport chain (ETC), which catalyses the oxidation of NADH and FADH_2_ to NAD^+^ and FAD respectively, with the associated reduction of molecular oxygen, to which electrons are transferred, to water. During the process, protons are translocated against a gradient in the intermembrane space by complexes I, III, and IV; the generation of a proton electrochemical potential (Δ*μ*_*H*_^+^), also called proton motive force (pmf), is achieved, driving the ATP synthesis, which is catalyzed as the final step by the F_1_F_o_-ATP synthase (Complex V). The supramolecular organization of the respiratory chain, with the F_1_F_o_-ATPsynthase localized to mitochondrial cristae, where a higher surface density of protons is realized, allows a better enzymatic performance of complex V. (b) Diagram of the structure of mammalian F_1_F_o_-ATPsynthase. We can divide the enzymatic complex into 4 principal subdomains: a catalytic headpiece (*α*_3_ *β*_3_), hosting the three catalytic sites for ATP synthesis (one in each *β* subunit), a proton channel (*ac*_8_) and two stalks, the central rotor (*γδε*) and the peripheral stator (*bd*(F_6_)OSCP) that link the first two subdomains together. While protons flow through the F_o_ channel from the intermembrane space into the matrix, a rotation of the stator inside the catalytic headpiece is induced, allowing a cyclic change in *β*-subunits conformation and the synthesis of ATP (N.B. Subunits A6L, *e*, *f*, and *g* are omitted in the scheme).

The mitochondrial F_1_F_o_-ATPsynthase is the smallest rotary motor in nature. It is a multisubunit complex (~5,000 aminoacid residues, with a mass of ~600 kDa) consisting of an intrinsic membrane domain, F_o_ (~1,500 aminoacids), and a globular catalytic domain, F_1_ (~3,500 aminoacids), which extends into the mitochondrial matrix (Figure 1). In mammals, the enzyme contains 15 different subunits, nine of which form the F_o_ domain (*a*, A6L, *b*, *c*, *d*, *f*, F_6_, *e*, and *g*), while the F_1_ domain is instead composed of only six (*α*, *β*, *γ*, *δ*, *ε*, and OSCP) [15–17]. The Inhibitory Factor 1 (IF_1_) is often regarded as the 16th subunit, although we will learn in this review that the protein is far more correctly defined as its endogenous regulator. 

The enzyme could be ideally divided into 4 principal subdomains: the catalytic headpiece (*α*_3_ *β*_3_), hosting the three catalytic sites for ATP synthesis (one in each *β*-subunit), the H^+^ channel (*ac*_8−15_) and two stalks, the central rotor (*γδε*) and the peripheral stator (*bd*(F_6_)OSCP), that link the first two subdomains together. The remaining minor subunits *e*, *f*, *g*, and A6L are associated with the proton channel; in particular, subunits *e* and *g* are involved in the dimerization of the complex [18] (a scheme of the structure of the ATP synthase is reported in Figure 1).

The crystal structure of the mitochondrial F_1_-ATPsynthase, extracted from bovine heart mitochondria, was revealed at the beginning of the Nineties [19, 20]. In this structure, F_1_ appears like a flattened sphere of 80 Å in height and 100 Å in width, with three *α*- and three *β*-subunits arranged alternately forming a cylinder around the coiled-coil structure of the *γ*-subunit. Sequences of *α*- and *β*-subunits are 20% identical [20], and this homology is reflected in their similar folds. All *α*-subunits have almost the same conformation, while the three *β*-subunits adopt three different tertiary structures and are in three diverse nucleotide-bound states: the first, named *β*_TP_, hosts the binding site for ATP; the second, called *β*_DP_, binds with high-affinity ADP and *P*_*i*_; the third, *β*_*E*_, does not efficiently bind any nucleotide. This asymmetry in the conformation and nucleotide occupancy of *β*-subunits supports the binding change mechanism of catalysis theorized by Boyer in the early 1970s, and fully developed in 1993 [21, 22]. According to this model, the three catalytic sites can be in three different conformations at any given time. *β*_TP_ and *β*_DP_ have, respectively, a “tight” (T) and a “loose” (L) form, in which ADP and *P*_*i*_ are converted into ATP, while the “open” conformation (O), adopted by *β*_*E*_, permits the release of the newly formed ATP and the acceptance of another molecule of ADP. ADP + *P*_*i*_ are theorized to enter into an O *β*-subunit, which then assumes an L conformation allowing the synthesis of ATP. After the synthesis, subunit *β* is in the T conformation, before assuming the O conformation in order to release ATP. The sequential interconversion between these different conformations, driven by the rotation of the central *γ*-subunit relative to the (*αβ*)_3_ subcomplex, enables the catalysis.

Unfortunately, there is less structural information on the F_o_ domain of ATP synthase than the F_1_ complex. In the ring, subunits *c*, whose structure was determined by both NMR spectroscopy [23, 24] and X-ray crystallography [25], folds as a hairpin composed by two transmembrane *α*-helices linked by a polar loop; part of the ring is in contact with subunit *a*, formed presumably by five transmembrane *α*-helices and containing two proton half-channels that do not span the membrane [26].

To briefly describe the functioning of the F_1_F_o_-ATPsynthase and its rotational catalysis, we must divide it into two parts: a central “rotor” (F_1_ *γε*-F_o_ *c*_10_) and a surrounding “stator” (F_1_ *α*_3_ *β*_3_ *δ*-F_o_ *ab*_2_). The driving force for the rotation is generated by the electron-transport chain (ETC) and is based on the magnitude of the proton electrochemical potential across the inner membrane; this gradient allows H^+^ to flow through the F_o_ domain (the *c*-ring) causing the rotation of the whole “rotor.” The precession of the *γ*-subunit, which contacts only one *β*-subunit at a time, induces cyclic changes in the “stator” so that ATP can be synthesized.

The rotational mechanism is extremely complex and depends on the structures of subunits *a* and *c*; the latter contain, in the middle of their *C*-terminal *α*-helix, a critical aminoacid residue, D61, which can either be in a protonated (lipophilic) or unprotonated (hydrophilic) form. Protonation and deprotonation of this aspartic acid residue is at the basis of the *c*-ring rotation dictating the membrane affinity of the single subunits. This occurs only in an aqueous environment and is realized when subunit *c* is in contact with subunit *a*, and the aspartate residue is hosted in one of the proton half-channels (see [27] for further details). In respiring mitochondria, the H^+^ motive force ensures the entrance of H^+^ residing in the intermembrane space into the cytosolic half-channel. The [H^+^] is more than 25 times higher on the cytosolic side than on the matrix side, and the ΔΨ_*m*_ of 140–180 mV increases the [H^+^] near the mouth of the cytosolic half-channel, thus resulting in protonation of the enclosed aspartate. Once the key residue is protonated, the *c*-ring can rotate clockwise (looking from the intermembrane space side), and the D61 of a new subunit *c* enters the matrix half-channel of subunit *a*, releasing a H^+^ into the mitochondrial matrix. Thus, the difference in [H^+^] and potential on the two sides of the membrane leads to different probabilities of protonation through the two half-channels, which yields directional rotational motion. The coupling between the rotation of the *c*-ring and the conformational changes in the (*αβ*)_3_ barrel is guaranteed by the tight link of the ring with the central stalk; while the *c*-ring rotates, the *γ*-subunit turns inside the *α*_3_ *β*_3_-hexamer, whose rotation is blocked by the presence of the peripheral stalk. Thus, the proton-gradient-driven rotation of the *c*-ring drives the rotation of the *γ*-subunit, which in turn promotes the synthesis of ATP through the binding-change mechanism. A highly conserved acidic cluster sequence in the *C*-terminal helical domain of the *β*-subunit (the DELSEED motif) is thought to be essential for ATP synthesis by coupling catalysis and rotation [28]. After the binding of an adenosine nucleotide in the catalytic site, the *C*-terminus is lifted up to the nearly immobile *N*-terminal part of the protein, and the *β*-DELSEED sequence is moved in contact with the *γ*-subunit, probably allowing the coupling of the *γ*-subunit torque with the rotation of the F_1_ domain (this hypothesis is still controversial [29]).

Each 360° rotation of the *γ*-subunit leads to the synthesis and release of three molecules of ATP. The number of subunits in the *c*-ring, which ranges from 8 (in bovine and probably in all vertebrates and invertebrates F_o_ domain [30]) to 15 (in *Spirulina platensis* [31]), determines the number of protons that are required to generate a molecule of ATP. 

## 3. Localization and Regulation of the Enzyme

Mitochondria are the sites where cellular energy is most abundantly produced, due to the constant activity of the mitochondrial F_1_F_o_-ATPsynthase. Although publications have suggested that it may also be localized on the plasma membrane [32, 33], we shall be exclusively discussing and referring to that embedded in the mitochondrial inner membrane.

As the F_1_F_o_-ATPsynthase is a reversible nanomotor, it can also hydrolyze ATP by translocating H^+^ from the matrix into the intermembrane space (an event that vigorously occurs when the enzyme is in isolation [34]). It does so to maintain the mitochondrial membrane potential (ΔΨ_*m*_) at a suboptimal level during deenergized conditions that occur when respiration is impaired by defects in the activity of the ETC, or when the mitochondrial inner membrane is leaky due to alterations in its structural integrity [35]. The ΔΨ_*m*_ is not only important for ATP production, but also for mitochondrial protein import and assembly [2]. Disruption of the ΔΨ_*m*_ is therefore implicated in various apoptotic phenomena (see [36]), being its maintenance crucial for cell viability.

The reversal of the F_1_F_o_-ATPsynthase is avoidable in eukaryotes and the enzyme must be controlled to prevent futile hydrolysis of ATP when the transmembrane proton electrochemical gradient collapses. Only facultative anaerobic bacteria employ this method for generating a vital proton electrochemical gradient in the absence of oxygen [37].

When reversal of the F_1_F_o_-ATPsynthase does occur, the depletion of cellular ATP can be more or less severe depending on the energy requests of the tissue, but in organs with high ATP demand, like brain or skeletal muscle, or in case of augmented ATP request, cellular demise is tangibly accelerated.

Apart from pathological states, repression or upregulation of ATP synthesis normally occurs in physiological conditions when intracellular ATP levels are, respectively, sufficiently high or too low. It has been calculated that, in eukaryotic cells, the rate of ATP utilization changes by a factor of 5–10 [38], such as during exercise and/or acclimatization.

Several are the mechanisms by which the activity of the F_1_F_o_-ATPsynthase is regulated: (a) *transcriptional factors* [39, 40]; (b) *translational control* [41–43]; (c) *modulation of the electron transport chain or the citric acid cycle* [44, 45]; (d) *ADP inhibition* [37] and (e) *regulatory proteins*, such as IF_1_. Although recent evidence has also suggested that the oncoprotein Bcl-XL interacts with the F_1_F_o_-ATPsynthase [46, 47], IF_1_ is the only molecular regulator of the enzyme characterized both biochemically and functionally. Nonetheless, other proteins which directly interact with the enzyme have been identified, such as factor B [48], essential for ATP synthesis and implicated in the regulation of the F_1_F_o_-ATPsynthase oligomerization, CaBI [49], which may up-regulate the enzyme in response to increased cytoplasmic Ca^2+^, and finally S100A1 [50], which has been found to enhance the enzymatic performance in cardiac muscle.

## 4. The Inhibitory Factor 1 (IF_1_) 

**Figure 2:**
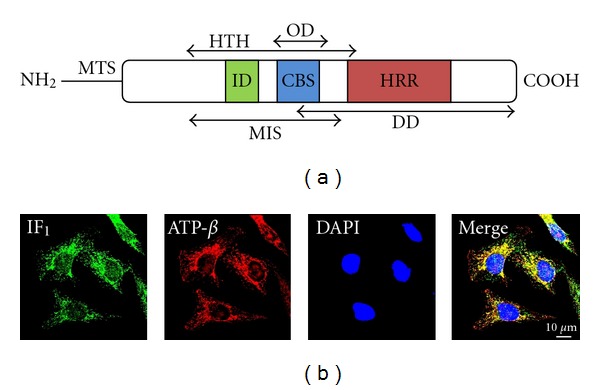
IF_1_: structure and intracellular localization. (a) Schematic representation of bovine IF_1_. The mature protein is composed of 84 residues and is **α**-helical along most of its length; an amine-terminal presequence of 25 aminoacids represents the mitochondrial targeting sequence (MTS) required for the trafficking of IF_1_ into the mitochondrial matrix. In complex, IF_1_ shows an ordered *N*-terminal region, which adopts a helix-turn-helix structure (HTH: residues 14–50) and is flanked by two disordered regions. The inhibitory domain (ID) is located at the *N*-terminus and is part of the minimal inhibitory sequence (MIS: residues 14–47) necessary for a correct interaction with the F_1_ domain of the ATP synthase. A calmodulin-binding site (CBS: residues 33–42) have been identified at positions 33–42, followed by a histidine-rich region (HRR: residues 48–70) which is implicated in the pH-sensing mechanism and hence in the dimerization. The dimerization of IF_1_ depends on the *C*-terminal region, which hosts the dimerization domain (DD: residues 37–84), while the oligomerization domain (OD: residues 32–44) is located in the *N*-terminal region of the protein, so that after oligomerization the inhibitory domain is hidden and the protein inactivated. (b) Immunocytochemical localization of IF_1_ in HeLa cells: the preferential mitochondrial matrix compartmentalization of the protein is shown by its colocalization with the ATP synthase. Cells were costained with anti-IF_1_ and anti-F_1_F_o_-ATPsynthase *β* chain antibodies, while DAPI was used for nuclear counterstaining.

IF_1_ was discovered in 1963 by Pullman and Monroy [6] in mitochondria from bovine hearts (a schematic representation of the structure of bovine IF_1_ is reported in Figure 2(a)). To date, IF_1_ homologues have been isolated from other mammals (e.g., rat [51] and human [52]), yeast (*Saccharomyces cerevisiae* [53] and *Candida utilis* [54]) and plants [55]. IF_1_ is a small, basic, heat-stable protein of approximately 10 kDa (in mammals the mature form of the polypeptide is composed of a number of amino acid residues which ranges from 81 in human, chimpanzee, dog and mouse, to 84 in cow). It is predominantly compartmentalized inside the mitochondrial matrix (Figure 2(b)), although studies have proposed that IF_1_ is also present in the cytosol and on the plasma membrane [56], as well as secreted into the extracellular environment, where it is implicated in the modulation of the activity of endothelial cells [32]. Intriguingly, in this very extramitochondrial localization, a role for hepatic HDL-cholesterol and triglyceride metabolism was also proposed [32, 57].

**Figure 3:**
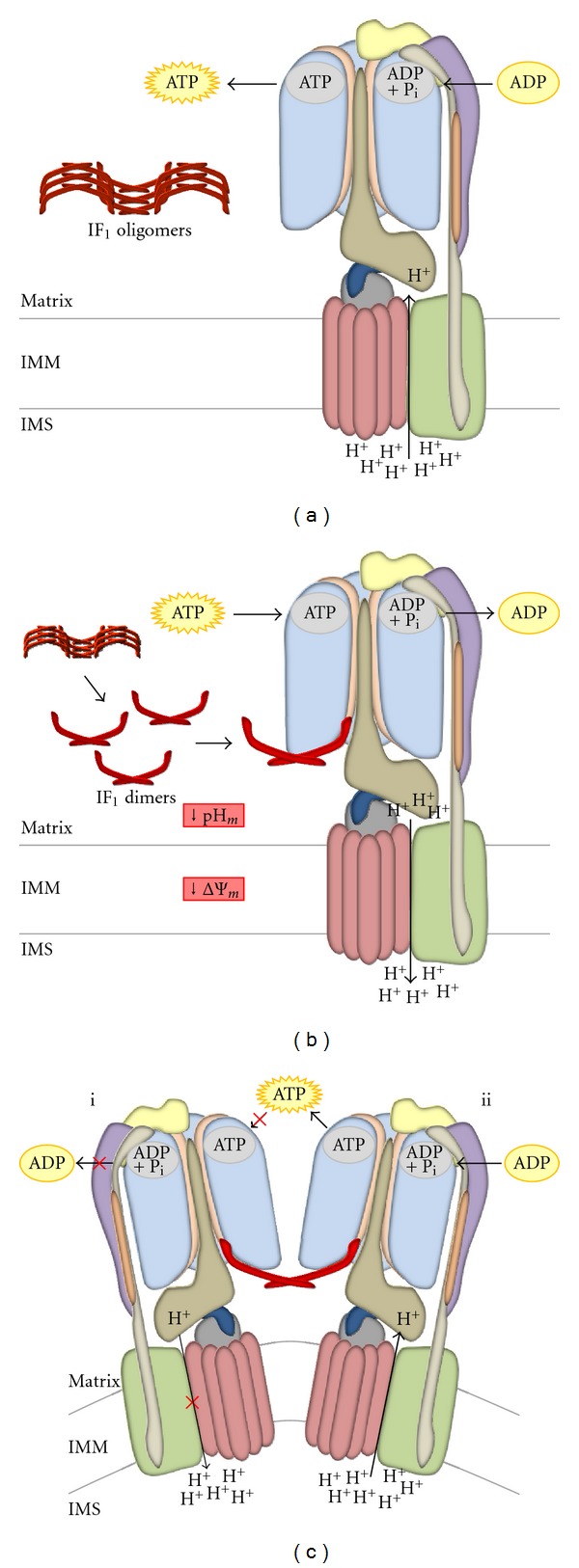
Interaction of IF_1_ with the F_1_F_o_-ATPsynthase. When mitochondria are in normal “energized” conditions (a), the F_1_F_o_-ATPsynthase can sustain physiological levels of ATP synthesis thanks to the presence of sufficient mitochondrial inner membrane potential; in this situation, the matrix pH is slightly basic, and IF_1_ is predominantly present in its inactive, oligomeric form. When the electrochemical H^+^ gradient is lost, the F_1_F_o_-ATPsynthase starts hydrolysing the ATP imported from the cytosol to pump H^+^ back into the intermembrane space (b), restoring ΔΨ_*m*_. The augmented [H^+^] in the matrix causes a fall in pH that induces the disruption of IF_1_ oligomers and the release of free active dimers. The binding of IF_1_ dimers at the interface between *α*- and *β*-subunits of the F_1_ domain is responsible for the selective inhibition of ATP hydrolysis (c–i), while its synthesis is not affected (c–ii). Active IF_1_ is able to interact with two F_1_ domains at the same time, inducing the dimerization of the F_1_F_o_-ATPsynthase (c), with subsequent increased enzymatic performance and cristae formation.

IF_1_ interacts with the catalytic subunit of the F_1_F_o_-ATPsynthase, inhibiting the hydrolysis of ATP under conditions that favour the reversion of the enzyme activity (Figure 3(b)). The regulatory protein is therefore an indispensable component to protect the cell from ATP depletion-driven damage and demise.

IF_1_ completely inhibits, through a noncompetitive mechanism, the ATP-hydrolyzing activity of the F_1_F_o_-ATPsynthase without affecting the synthesis of ATP during oxidative phosphorylation, although a few studies argue differently on this [58, 59]; nevertheless, IF_1_ is reported to be largely active only at low pH [60], hence in conditions of ATP hydrolysis. The inhibitory protein binds to the soluble F_1_ domain in a 1 : 1 stoichiometry in the presence of Mg^2+^ and ATP [61]. This is important for maintaining cellular ATP, by preventing its hydrolysis, when the H^+^ electrochemical gradient across the mitochondrial inner membrane is lost (e.g., during hypoxic/ischaemic conditions) and the enzyme reverses its activity to transiently restore ΔΨ_*m*_ [58, 59].

IF_1_ is encoded by the *ATPIF1 *gene, localized at chromosome 1, and is synthesized as a propolypeptide (106 residues in humans [52]) harbouring a highly conserved*N*-terminal presequence of 25 amino acids (identified by the comparison between the amino acid sequences deduced from the cDNA and the purified protein) presumably important for the mitochondrial targeting of the protein [61]. The mature polypeptide (81–84 aa in mammals) is significantly conserved among various species. The human protein exhibits 67–74% of sequence homology with other known mammalian IF_1_ inhibitors, and also partial homology with the yeast inhibitor Inh1p [52]. Interestingly, there is a strong correlation between the high sequence conservation and function, as IF_1_ from one species is able to inhibit the F_1_F_o_-ATPsynthase from another, including yeast [51, 62, 63]. Conversely, yeast IF_1_ is not able to inhibit the animal F_1_ domain because its activity is stabilized by accessory proteins which have no homologues in animals [64].

The most conserved segment of the inhibitory factor is the region that interacts with the F_1_F_o_-ATPsynthase, comprising amino acid residues 20–50 (90% identity between the human and bovine sequences, 80% between bovine and rodent [61]), and a lot of research into understanding the minimal inhibitory sequence of IF_1_ has focused on this sequence. Van Raaij and coworkers investigated this [65], by measuring the activity of several truncated forms of the protein. The intact and truncated forms assayed for inhibition of F_1_F_o_-ATPsynthase using IF_1_-depleted submitochondrial particles revealed that the minimal inhibitory sequence consists of residues 14–47.

In 2001, Ichikawa and colleagues [66] showed by amino acid replacement that in yeast five residues (F17, R20, R22, E25 and F28) are essential for the inhibitory activity of the protein. Two years later it was brought up that six homologous residues of bovine IF_1_ (F22, R25, E26, Q27, E30, and Y33), which form a cluster on the surface of the *α*-helix [67], represent the inhibitory site of the protein [68].

## 5. IF_1_: Molecular Structure and Conformational Changes

The molecular structure of IF_1_ was initially characterized by Cabezon et al. in 2001 [67] (2.2 Å resolution X-ray crystallographic analysis). The protein is *α*-helical along most of its length (~90 Å [69]) and is active as a dimer at low pH (<6.7). The dimerization of two IF_1_ monomers involves their *C*-terminal regions (residues 37–84 [70]), which form an antiparallel double-stranded coiled-coil unit stabilized by complimentary hydrophobic interactions between the two helices, involving residues 49–81 [67]. Within the dimer, the two minimal inhibitory sequences are at the opposite ends (the dimer shows an end-end distance of at least 130 Å, while the distance between the two inhibitory regions is 62 Å) and can react with two F_1_ domains simultaneously (Figure 3(c)). When the pH is above neutrality (7.0-8.0), the dimers can assemble inactive tetramers or higher oligomers by forming antiparallel coiled-coils in the *N*-terminal regions (residues 32–44 are involved [70]). Every protomer of the dimer can participate in two coiled-coil units with two different helices, binding two dimers simultaneously. The formation of the oligomers masks the inhibitory sequences of the dimers, so that IF_1_ cannot bind the F_1_F_o_-ATPsynthase (Figure 3(a)). Dimers and oligomers are in equilibrium at pH 6.5 [70].

Mammalian IF_1_ contains five highly conserved histidines (at positions 48, 49, 55, 56 and 70) that, if chemically modified or replaced, lead to a complete loss of the pH-susceptible activity of the protein without affecting its inhibitory capacity [60, 71]. This histidine-rich region (residues 48–70) is involved in the pH sensing mechanism of bovine IF_1_, and undergoes conformational changes depending on acidity or alkalinity of the environment [61]. Critical for the pH-dependent interconversion between the two aggregation states of the polypeptide is the histidine 49 [70, 71].

It was observed that replacement of this residue with a different amino acid induces full activation of IF_1_ at pH 8, and abolishes the ability of the dimers to form oligomers [67, 71]. The five histidines seem to be important for the pH-regulated decrease in activity between pH 6.7 and 8.0, even if they may not represent the only mechanism responsible for such regulation. In fact, a pH-dependent activity was also observed in the IF_1_ 22–46 peptide [72] and detected in a 12-residue segment from 32 to 43 [70]; moreover, H49 is not conserved in yeast, suggesting a diverse pH-sensitivity of the protein [73].

Apart from controlling the oligomerization of the polypeptide and consequently the availability of its inhibitory site, the pH itself was proposed to represent the switch between inactive and active forms by controlling the helical content and the flexibility of the whole protein [74]. At low pH (~6.7), the helical content seems to be lower, so that the *N*-terminal region is less ordered and, instead of forming a coiled-coil unit with two other dimers, assumes the correct conformation for binding the F_1_ complex. However, this theory is controversial since pH was thought to simply act by modulating the electrostatic interactions between the polar residue-enriched *N*-terminal regions of the dimers, as presented by Cabezon et al. [67]. Recently, Ando and Ichikawa [73] discovered that pH could effectively change the conformation of the active site by acting on a highly conserved glutamate residue, E26 in bovine IF_1_ or E21 in yeast IF_1_ (H49 is not conserved in yeast and, as a consequence, cannot represent the only pH sensor residue of the protein). The mechanism of pH-dependency mediated by glutamate regulates only the inhibitory activity of the F_1_-binding site and not the aggregation state of the polypeptide.

Very little is known about the transcriptional and posttranscriptional regulations of IF_1_, despite speculations on a possible contribution by the hypoxia-inducible factor 1-*α* (HIF1-*α*) [75] and evidence for a downregulation mediated by the Immediate early response gene X-1 (IEX-1) [76].

## 6. IF_1_ in Complex with the F_1_F_o_-ATPsynthase: Outcome on Mito-Ultrastructure 

The molecular crystal structure of bovine IF_1_:F_1_-ATPase complex is available [69, 77]. In the complex, IF_1_ shows an ordered *N*-terminal region and a disordered *C*-terminal part. The former adopts a helix-turn-helix structure, in which the two *α*-helices extend between residues 14–18 and 21–50 and are linked by a turn from residues 19-20; residues 4–37 are directly involved in binding the F_1_ domain. Residues 4–18, which are disordered in the dimer, are instead resolvable after binding.

In the bound form, the inhibitory sequences of the two protomers in the dimer become closer (their distance is shortened from 62 Å to 31 Å). This is possibly due to the flexibility of the *C*-terminal coiled coil region, which probably has a greater curvature in the complex. The long helix at the *N*-terminal region of IF_1_ is inserted almost completely into the F_1_ domain; only residues 47–50 lie outside of it. This points to the central axis of the *γ*-subunit and forms a ~45° angle with it, heading from the external surface towards the central cavity.

The contacts between the bovine inhibitor and the inhibited F_1_ domain are essentially located at the interface between the *α*_DP-_ and *β*_DP-_ subunits, even though IF_1_ also contacts small portions of the *γ*−, *α*_*E*_− and *β*_TP-_ subunits [77]. Although the inhibitory sequence is comprised of residues 14 to 47, this is not the only region that interacts with the F_1_F_o_-ATPsynthase. In fact, as demonstrated by the time-dependent loss of inhibition seen by van Raaij and co-workers [65], residues 1–13 and 48–56 are important for stabilizing the structure (the first peptide interacts directly with the F_1_ domain, while the second probably contributes to stabilize only the IF_1_ dimer).

With different approaches, Cabezon et al. [77] and Ichikawa et al. [66] found that a cluster of six residues, F22, R25, E26, Q27, E30 and Y33 in bovine IF_1_, is essential for the inhibitory activity of the peptide. In the crystal structure of the IF_1_:F_1_ complex, the three core residues of the cluster, F22, E26 and E30, interact with three highly conserved residues of *β*_DP_ (*β*R408, *β*R412, and *β*E454) which are essential for the enzymatic catalysis and regulation of the F_1_F_o_-ATPsynthase [78]. E30 directly interacts with the *β*-subunit of F_1_ and seems to be essential for the F_1_-IF_1_ interaction (Figure 3). Interestingly, one of the three residues, E26, is also implicated in the pH sensing mechanism of IF_1_. In 2008, Ando and Ichikawa [73] discovered the key role of the glutamate residue in the pH-dependent activity of the inhibitory protein. They proposed that it was the high pH, by inducing the dissociation of the carboxyl group of E26, to affect the conformation or direction of the side chain of the neighboring residue E30, thus destabilizing the interaction between IF_1_ and the F_1_ domain.

As proposed by Walker and co-workers [69], IF_1_ seems to inhibit the hydrolysis of ATP that occurs in mitochondria under hypoxic conditions by initially binding to the *α*_*E*_/*β*_*E*_-interface of the F_1_ domain, an event that appears to require the presence of ATP in the active site. It was also suggested that the binding of ATP induces a conformational change in the *β*_*E*_-subunit, which creates the binding site for IF_1_ [77].

Garcia and colleagues provided the first compelling evidences for a role of IF_1_ in promoting the dimerization of the F_1_F_o_-ATPsynthase, and for its involvement in the biogenesis of mitochondrial cristae [79]. Moreover, they also showed that, by increasing the expression of IF_1_ in rat liver or AS-30D hepatoma mitochondria, a rise in the dimer/monomer ratio of the F_1_F_o_-ATPsynthase (correlated with an increase in the enzyme activity) is obtained, while the removal of the inhibitory protein from rat liver or bovine heart mitochondria resulted in a reduction of that ratio [8]. The dimerization of the enzyme is essential for a correct biogenesis of mitochondrial cristae; in fact, it represents a prerequisite for the generation of larger oligomers with a ribbon-like structure that promotes curvature and growth of tubular cristae membranes [80].

In a recent study, we demonstrated the pivotal role of IF_1_ in cell physiology through promotion of the F_1_F_o_-ATPsynthase dimerization. Briefly, we showed that IF_1_ overexpression efficiently increase the activity and the ratio of dimeric to monomeric forms of the F_1_F_o_-ATPsynthase, with augmented cristae number, mitochondrial membrane stability, and mitochondrial volume [81], thus ensuring a correct mitochondrial inner structure. This is a phenomenon of secure relevance for apoptosis.

## 7. IF_1_ in Cell Pathology: from Limitation of the Mitochondrial ATP Consumption to Anaemia

Decreased ΔΨ_*m*_ induces the reversion of the F_1_F_o_-ATPsynthase, which starts hydrolysing ATP in the attempt to restore the H^+^ gradient through the mitochondrial inner membrane, transforming mitochondria in ATP consumers (Figure 3(b)). This condition associates with ischaemia, in which the interruption of tissue blood flow causes a reduction of cell oxygenation (hypoxia) inhibiting mitochondrial respiration. Clear evidence for the reversal of the F_1_F_o_-ATPsynthase during ischaemia can be obtained by using an ETC inhibitor (e.g., rotenone, which acts on complex I, or NaCN, which inhibits complex IV) and concomitantly adding oligomycin, an antibiotic that blocks the F_1_F_o_-ATPsynthase. These cells will experience a reduced depletion of ATP compared to those bathed with the ETC inhibitor but without oligomycin.

The negative effect of the reversal of the F_1_F_o_-ATPsynthase is coupled to the reversal of the adenosine nucleotide translocator (ANT) [82], an IMM transmembrane complex that, in physiological conditions, mediates the exchange of cytosolic ADP and mitochondrial matrix ATP (utilizing the different gradients between the two compartments). Reverse activities of both F_1_F_o_-ATPsynthase and ANT transform mitochondria from ATP producers to ATP consumers, leading to massive cytosolic ATP depletion in hypoxic cells following ischaemia.

Reduced intracellular ATP level is flanked by elevated cytoplasmic H^+^, Na^+^, and Ca^2+^ concentrations, inducing osmotic loading and mitochondrial/endoplasmic reticulum injury so that, during ischaemia, death of hypoxic cells by necrosis easily occurs.

Early reperfusion minimizes the extent of cellular damage, salvaging cells within ischaemic regions from necrosis, but it can also causes lethal injury to cells with severe ischaemia-induced metabolic derangements (reviewed in [83]). In the latter case, reperfusion alters the activity of plasma membrane transporters (e.g., abolishing acidosis-mediated inhibition of the Na^+^-Ca^2+^ exchanger and inducing the activation of calpain, which disturbs Na^+^-K^+^ pump function), thus leading to massive influx of Ca^2+^ into the cytosol and mitochondrial Ca^2+^ overload. At the same time, resupply of oxygen to mitochondria restores ATP production but also induces a rise in reactive oxygen species (ROS) production. Both mitochondrial Ca^2+^ overload and augmented ROS levels represent the prelude to the opening of the mitochondrial permeability transition pore (mPTP) and cell death.

The entity of the reversal of the F_1_F_o_-ATPsynthase and its action as an H^+^ motive ATPase during oxygen deprivation were shown by Jennings et al. in 1991 [7]. Studying the changes in ATP depletion and anaerobic glycolysis in totally ischaemic dog heart after inhibiting the F_1_F_o_-ATPsynthase via oligomycin, the same authors have evaluated that about 35% of ATP utilization during the first 90 minutes of total ischaemia in the dog heart is due to the reversion of the enzyme activity.

Oligomycin-mediated inhibition slows down ATP depletion during ischaemia. Rouslin and colleagues [11] proved that the antibiotic has a very small and transient effect on mitochondrial function when used in fast heart-rate animals, like rats, if compared to slow heart-rate species, like larger mammals are. The same authors have subsequently shown that this diversity depends on the different F_1_F_o_-ATPsynthase: IF_1_ ratios, with a diverse ability to inhibit mitochondrial-driven consumption of ATP when needed [84].

The estimation was that IF_1_ reduces the ATP-hydrolysing activity of F_1_F_o_-ATPsynthase during ischaemic conditions by up to 70–80% (in slow heart-rate species) [85], thus preventing cellular damage due to ischaemic conditions and delaying cell death when oxygen and glucose are limited. Upon reperfusion, the binding of IF_1_ to the F_1_F_o_-ATPsynthase is quickly reversed [86], so that sublethal ischaemic episodes could be followed by a relatively rapid recovery of intracellular ATP.

Although this model is challenged by later evidence showing that in rat heart, during ischaemic preconditioning, mitochondrial ATP hydrolysis is inhibited probably as a consequence of the binding of IF_1_ [87], the variations in ratio between the enzyme and its controller among animal species are still a fascinating possibility. Nonetheless, this ratio differs per se among organs and cell types of the same organ [12].

By modulating the expression of IF_1_ in human (HeLa) and murine (C2C12) cells, we demonstrated that, when IF_1_ is overexpressed, cells show a decrease in ATP consumption [81]. Thus, variations in IF_1_ expression could influence cellular or tissue resistance to ischaemic injury in different species or cell types.

### 7.1. Central Nervous System

Notably, IF_1_ expression is elevated in highly oxidative cells, like neurons and kidney proximal tubules [12], which are highly susceptible to mitochondria deregulations. In the central nervous system, for example, neurons and astrocytes show a great difference in the IF_1_ : F_1_-*β*-subunit ratio, which is ~1.45 in the former and ~0.8 in the latter; as a consequence, the inhibition of respiration with NaCN causes a progressive loss of ΔΨ_*m*_ in neurons, while in astrocytes the proton gradient is maintained at a new steady state [81]. Thus, higher levels of IF_1_ could be advantageous in cells highly depending on oxidative phosphorylation by preventing ATP depletion and quick cellular damage during ischaemia.

### 7.2. Preconditioning

A final interesting aspect is the highly probable involvement of IF_1_ in the ischaemic preconditioning mechanism. This phenomenon, which is characterized by the acquirement of a strong resistance to ischaemia in tissue undergoing brief, repeated periods of sublethal ischaemia, is commonly observed in heart, skeletal muscle and brain [61]. It is described as a slowing of energy metabolism with a decreased rate of ATP depletion during ischaemia [88]. IF_1_ is proposed to take part in this process after the observation that rat heart preconditioning associates with the inhibition of mitochondrial ATP hydrolysis during ischaemia [87]. This was later confirmed by Penna et al., who showed that decrease in the enzyme's activity after ischemic preconditioning correlates with an augmented binding of IF_1_ [89]. Moreover, a consensus exists that the opening of mitochondrial ATP-sensitive K^+^ channels plays a central role in cell protection during ischemic preconditioning, causing mitochondrial deenergisation and acidification owing to H^+^/K^+^ exchange [86]; both effects are likely to promote the binding of IF_1_ to the ATP synthase.

### 7.3. Cancer

The importance of mitochondrial metabolism in cancer cells is underlined by the frequently observed, close interaction of glycolytic enzymes with mitochondria. This creates a mutually sustaining relationship between glycolysis, which represents the primary metabolic pathway for tumours sustenance [90], and oxidative phosphorylation.

Regarding the F_1_F_o_-ATPsynthase endogenous regulator, IF_1_, its overexpression has been observed in many human carcinomas (including lung, colon, breast, and cervix carcinomas [10], Ehrlich ascites carcinoma [91], Zajdela hepatoma and Yoshida sarcoma [92]), but little is still known about the associated effects, and the few theories that have been put forward are highly controversial. Increased expression of the protein is associated with a higher binding efficiency to the F_1_F_o_-ATPsynthase [93], suggesting a greater protection of cancer cells against energy dissipation upon F_1_F_o_-ATPsynthase reversal. This was theorized by Chernyak et al. [91], although more recent work has cast doubt on this hypothesis, revealing a relationship between the inhibitor overexpression in human carcinomas and an increase in both ΔΨ_*m*_ and glycolytic rate [10].

The protein may therefore be involved in protecting tumour cells from cytosolic ATP depletion and excessive reactive oxygen species (ROS) production (the majority of tumours have little or no vascularization, so that cancerous cells grow in a hypoxic environment). Over and above that, to guarantee cell viability, mitochondria should not become ATP consumers. ATP depletion, ROS imbalance, low cytosolic pH and oxidation of NAD(P)H facilitate the opening of the mitochondrial permeability transition pore (mPTP) and the activation of the intrinsic apoptotic pathway [94]. Numerous studies have also demonstrated that transient hyperpolarization of the mitochondrial membrane can lead to cell apoptosis [38, 95]. By preventing ROS production and IMM hyperpolarization, IF_1_ could also protect cancer cells from ROS-mediated apoptosis. IF_1_ induces the dimerization of the F_1_F_o_-ATPsynthase [8], which might play an essential role in preventing both mitochondrial network fragmentation and cytochrome *c* release from mitochondrial cristae, thus inhibiting the activation of the intrinsic apoptotic pathway. Our previous studies seem to support these hypotheses [81], and, future and focused studies will shed light on this (Tan et al., *under review*). Moreover, an original recent work by Cuezva and co-workers has elegantly demonstrated that IF_1_ is protective against chemotherapy and supports cell proliferation of cancerous cells via the NfkB pathway [96]. Although this work focuses principally on the postmitochondrial effects of IF_1_, it is anyway a compelling evidence for a contribution to neoplastic degeneration and resistance to apoptosis. A starting point to unravel how mitochondrial structure and function are primed by IF_1_ overexpression, and to understand to what extent this dictates cellular transformation.

### 7.4. Luft's Disease

To date, the absence of IF_1_ has been correlated with only one human pathological condition of neuronal origin: a mitochondrial myopathy called Luft's disease, characterized by nonthyroidal hypermetabolism and densely packed mitochondrial cristae (it is one of the rarest of the mitochondrial diseases, with only two reported cases). Basal ATPase activity in one of the two patients was seven times higher than normal [13], and no IF_1_ activity was detected in fibroblasts cultured from the skeletal muscle [14]; however, no mutations in the *ATPIF1* gene were identified, and the genetic cause of the disease remains obscure.

### 7.5. Anaemia

Despite what discussed above, we have very recently collected evidences for a deficiency in the *ATPIF1* gene associated with a form of hypochromic anaemia (Shah et al., *under review*). The mechanism we propose is related with the secondary effect that the absence of IF_1_, and the consequent lack in its inhibitory activity on the F_1_F_o_-ATPsynthase reversion, has on ΔΨ_*m*_ and matrix pH. It is known that erythroids' differentiation is triggered by a decrease in ΔΨ_*m*_, that is responsible for a critical redistribution of intracellular Ca^2+^ and a transient activation of caspases [97]. Anyway, we found that the increase in mitochondrial matrix pH, which is observed in zebrafish models and murine cells carrying the mutated form of the *ATPIF1* gene, is causally linked to a decrease in ferrochelatase activity, which leads to defects in the incorporation of ^59^Fe into protoporphyrin IX to generate the hemoglobin prosthetic group heme.

Such a remarkable finding puts IF_1_ amongst the regulators of heme biosynthesis, not only describing a new mechanism for sideroblastic anaemia, but also confirming the involvement of the inhibitory protein in human pathologies related to mitochondrial disorders.

## 8. Conclusions

The F_1_F_o_-ATPsynthase is a wonderful machinery, with the unique capacity of producing and consuming energy, if necessary, to preserve the integrity of the organelle to which it belongs. A precise and sustainable way to regulate its activity is therefore paramount, and the Inhibitory Factor 1, a protein encoded by the nuclear DNA, represents the molecule deputed to do so. In the face of a well-defined biochemistry, its role in cell physiology and mitochondrial anatomy has been only recently discovered, posing the protein at the cross-road between dynamics and energy balance. This, together with growing evidence for a contribution to cell and tissue pathology, leads to novel ways to investigate and thoroughly address IF_1_ functional biology.
